# Supplementary material for: De novo biosynthesis of the hops bioactive flavonoid xanthohumol in yeast
Source: Nat Commun. 2024 Jan 4;15:253. doi: 10.1038/s41467-023-44654-5 (PMC10766616; doi:10.1038/s41467-023-44654-5)
Supplement: Supplementary file 1 — Supplementary Information [file 41467_2023_44654_MOESM1_ESM.pdf]

De novo biosynthesis of the hops bioactive flavonoid xanthohumol in yeast

Yang *et al.*

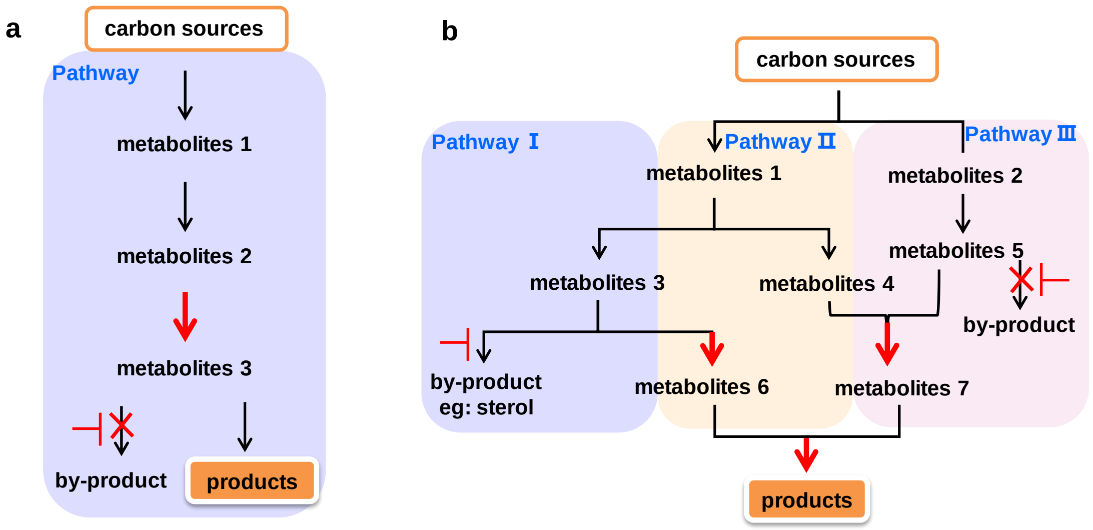

Supplementary Fig. 1. Comparing the single-channel and parallel biosynthetic pathways for natural product biosynthesis. a, A single metabolic pathway from carbon sources, which significantly increase the production of target product by simply strengthening rate-limiting steps and blocking side reactions. b, Parallel biosynthetic pathways require balancing the parallel modules for enhancing product biosynthesis, which were challenging.

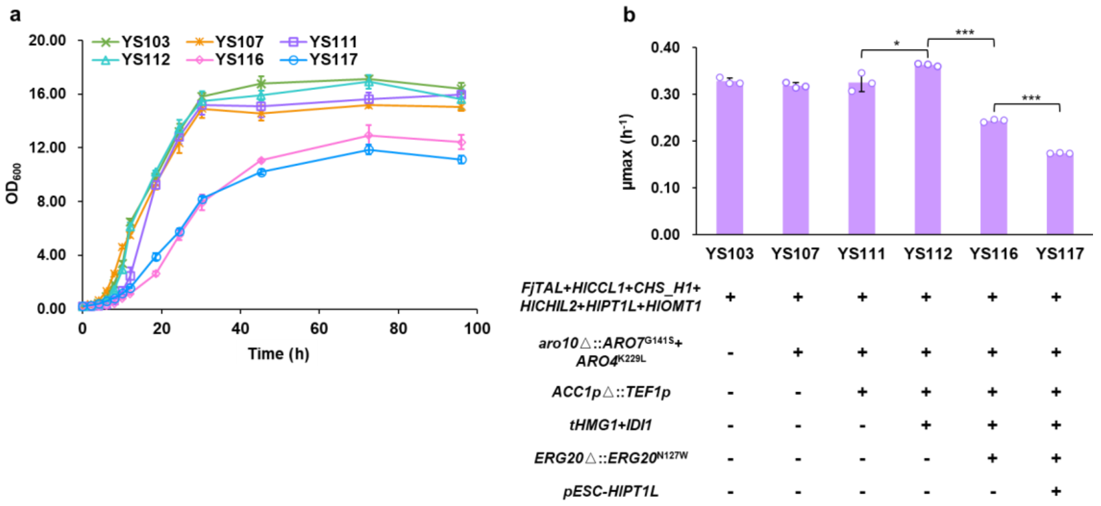

Supplementary Fig. 2. The effect of engineering the three parallel biosynthetic pathways on the cell growth. a, Growth curves over the batch cultivation in shake flasks. b, Maximum specific growth rates (μ_{max}). All strains were cultivated in 100 mL shake flasks containing 20 mL of minimal medium. Mean values ± standard deviations are shown (n=3 independent biological samples). One-tailed Student's *t*-test was used for comparing two groups ($*p < 0.05$, $**p < 0.01$, $***p < 0.001$), and *p* values were shown in b. Source data are provided as a Source Data file.

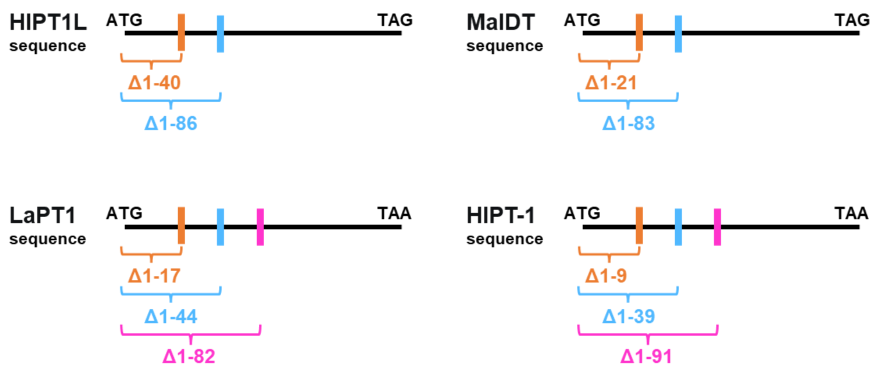

Supplementary Fig. 3. Schematic diagram of the truncated sequence positions of MaIDT, HlPT-1, LaPT1 and HlPT1L. Δ1-x represented the length of the sequence truncated from the N-terminal.

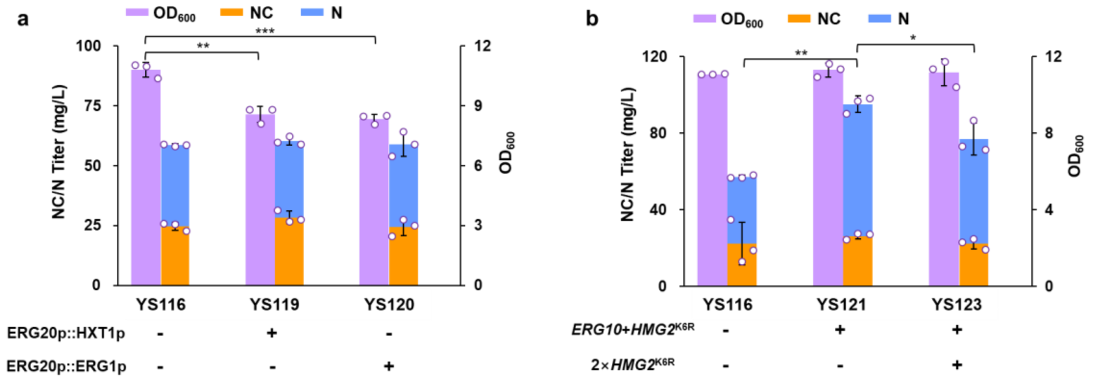

Supplementary Fig. 4. Engineering substrate supply for the production of precursors NC/N. a, Replacing the native promoter of ERG20^{N127W} with the promoter P_{HXT1} or P_{ERG1} failed in improving NC/N production, but hindered cell growth. b, Overexpression of ERG10 and HMG2^{K6R} increased NC/N production. However, overexpression of another two copies of HMG2^{K6R} decreased NC/N production. All strains were cultivated in 100 mL shake flasks containing 20 mL of minimal medium. Mean values ± standard deviations are shown (n=3 independent biological samples). One-tailed Student's *t*-test was used for comparing two groups (*$p < 0.05$, **$p < 0.01$, ***$p < 0.001$). Source data are provided as a Source Data file.

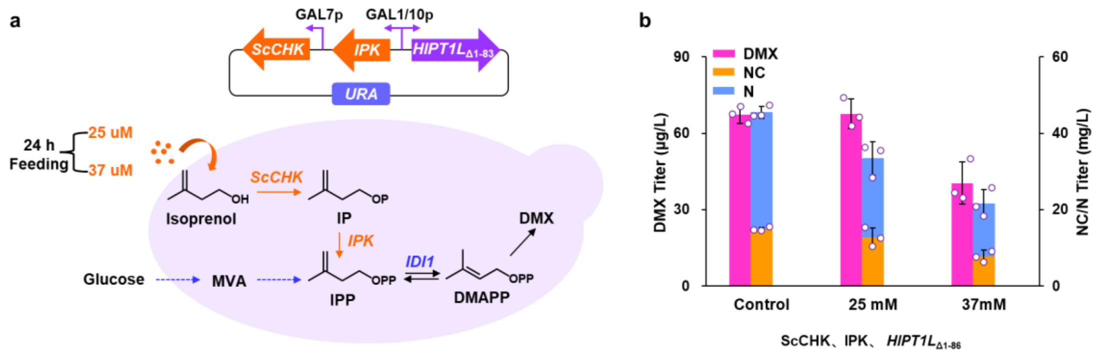

Supplementary Fig. 5. Engineering IUP pathway for DMX production. a, Schematic diagram of overexpression of the exogenous IUP pathway in strain YS116. The high copy plasmid pESC-URA was used to overexpress the key genes *ScCHK*, *IPK* and *HlPT1L*$_{\Delta 1\text{-}83}$. 25 µM or 37 µM isoprenol were fed at 24 h strain cultivation. ScCHK, choline kinase from *S. cerevisiae*; IPK, isopentenyl phosphate kinases; IP, isopentenyl monophosphate. IPK from *Arabidopsis thaliana* were codon-optimized for *S. cerevisiae*. b, IUP bypass pathway could not increase the DMX production. All strains were cultivated in 100 mL shake flasks containing 20 mL of minimal medium. Mean values ± standard deviations are shown (n=3 independent biological samples). Source data are provided as a Source Data file.

a

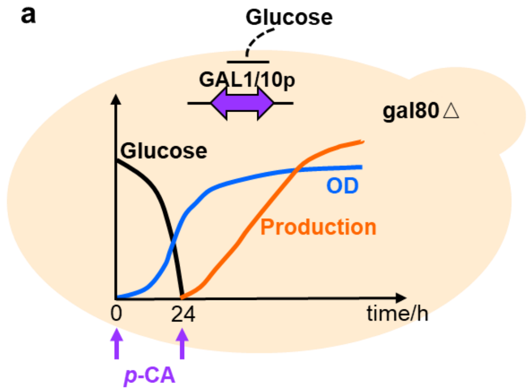

b

	Feeding time (h)	Feeding *p*-CA Concentration (mg/L)
Control	0	0
0	0	140
24	24	140
0+24	0, 24	140,140
24 (double)	24	280

Supplementary Fig. 6. Feeding different concentrations of *p*-CA at different times under the guidance of GAL regulation system. a, Schematic diagram of dynamic regulation of product biosynthesis using GAL regulation system. Deleting GAL80 (gal80Δ) enabled repression of the P_{GAL}-controlled genes at high glucose concentration and high-expression at low glucose concentration (production phase). $P_{GAL1/10}$ was a bidirectional promoter. *p*-CA was fed at 0 h or 24 h. b, The feeding concentration and feeding time of *p*-CA were shown in the table.

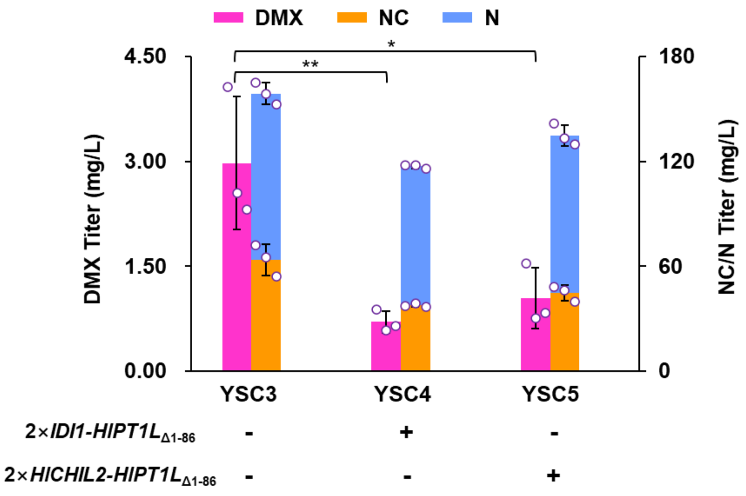

Supplementary Fig. 7. Overexpression of IDI1-HlPT1L$_{\Delta 1\text{-}83}$ or HlCHIL2-HlPT1L$_{\Delta 1\text{-}83}$ fusion in strain YSC3 decreased the production of DMX and NC/N. The fusions were constructed by using a flexible linker $(GGGS)_3$. All strains were cultivated in 100 mL shake flasks containing 20 mL of minimal medium. Mean values ± standard deviations are shown (n=3 independent biological samples). One-tailed Student's *t*-test was used for comparing two groups (*$p < 0.05$, **$p < 0.01$, ***$p < 0.001$). Source data are provided as a Source Data file.

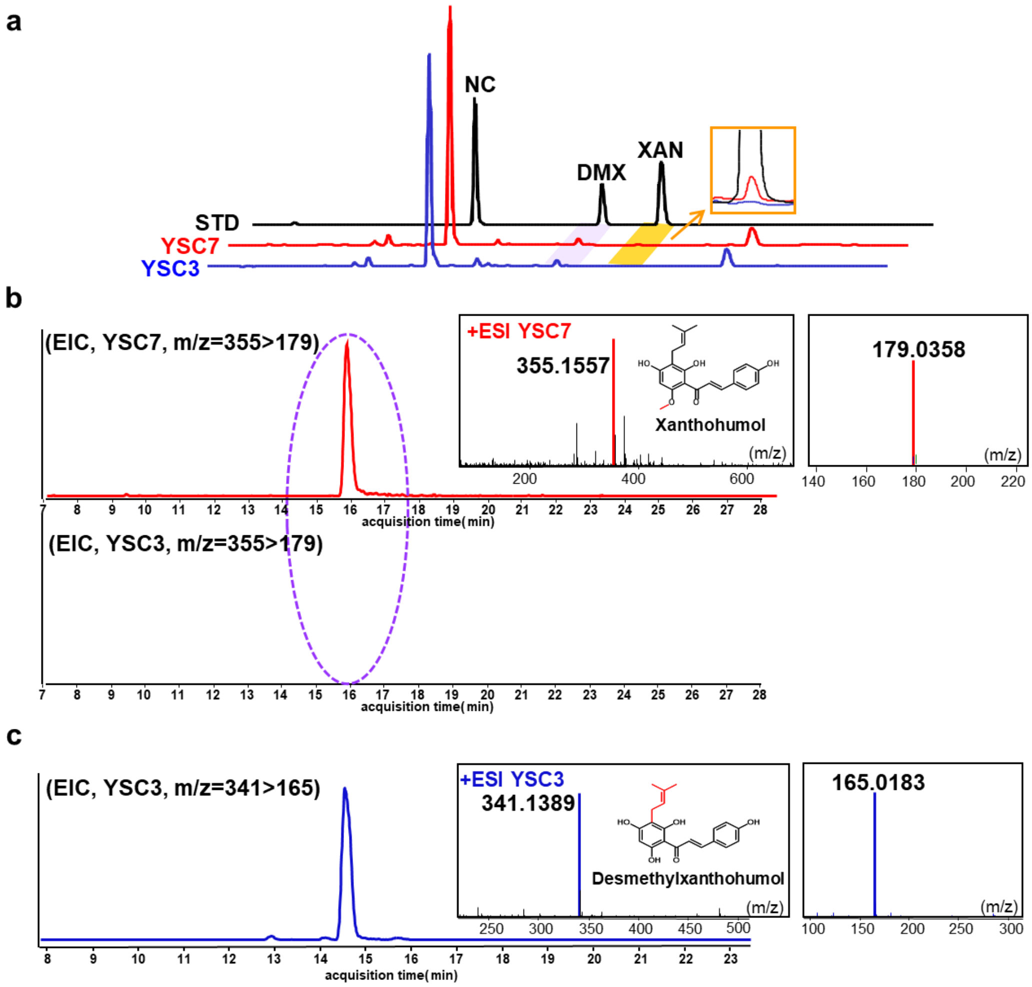

Supplementary Fig. 8. LC/MS-MS analysis of xanthohumol (XAN) and DMX in control strain YSC3 and strain YSC7 obtained using positive ion electrospray, collision-induced dissociation and selected reaction monitoring. a, HPLC analysis of the xanthohumol standard, the fermented product of the xanthohumol-producing strain YSC7, and the control strain YSC3. b, LC/MS-MS analysis of the control strain YSC3 and the engineered strain YSC7 showed that xanthohumol was synthesized in strain YSC7. Chromatogram and mass spectra of selected ions of m/z 355.1557 > 179.0358 for xanthohumol. c, LC/MS-MS analysis of DMX in the strain YSC3. Chromatogram and mass spectra of selected ions of m/z 341.1389 > 165.0183 for DMX. Source data are provided as a Source Data file.

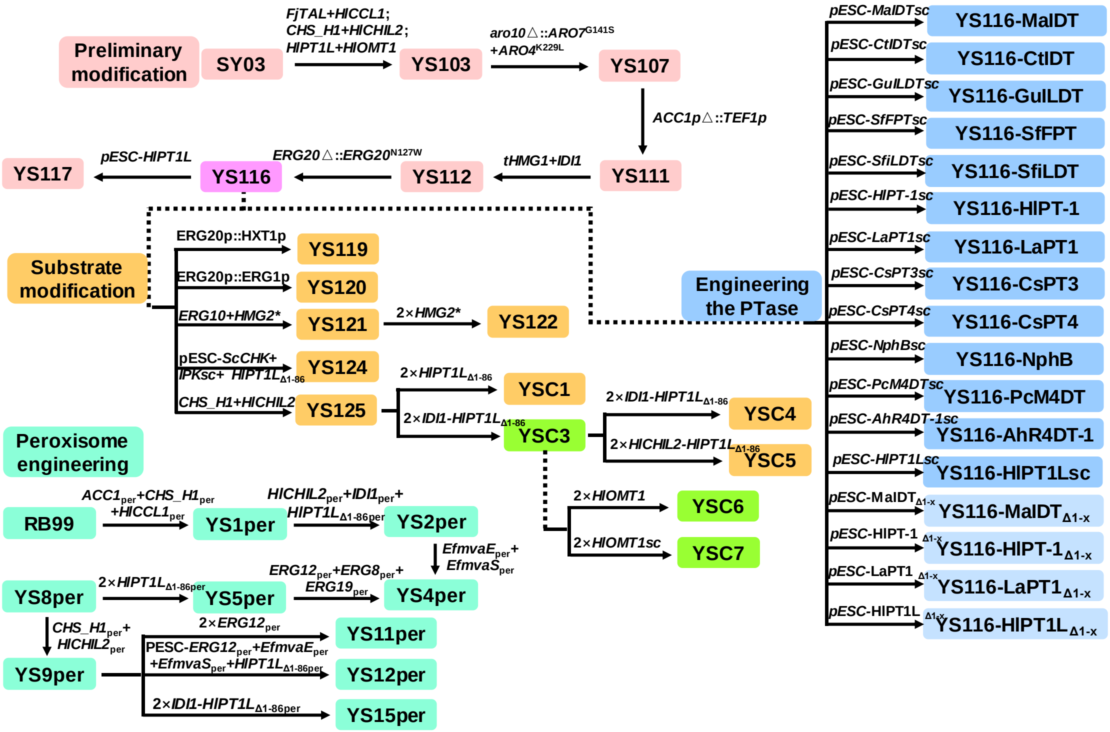

Supplementary Fig. 9. Flowchart of yeast strain construction in this study.

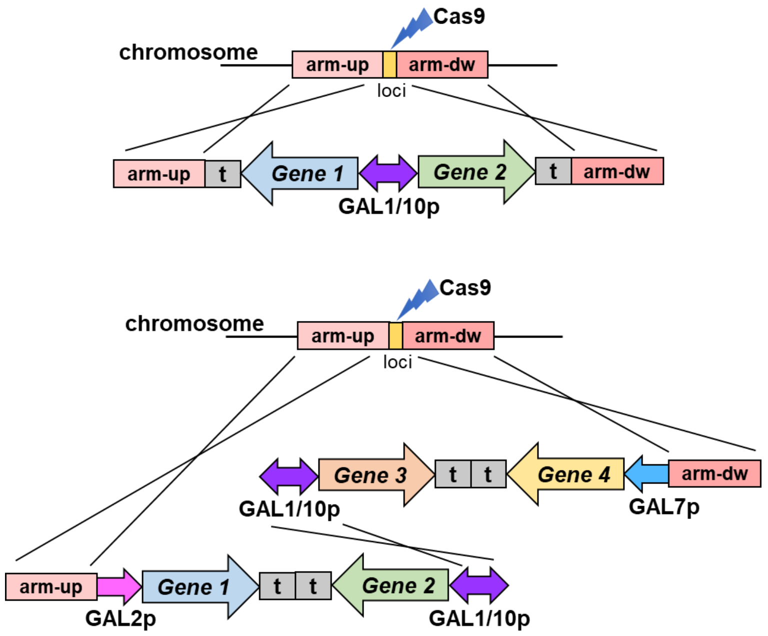

Supplementary Fig. 10. The types of donor DNAs used for strain construction.
