# Supplementary material for: De novo biosynthesis of the hops bioactive flavonoid xanthohumol in yeast
Source: Nat Commun. 2024 Jan 4;15:253. doi: 10.1038/s41467-023-44654-5 (PMC10766616; doi:10.1038/s41467-023-44654-5)
Supplement: Supplementary file 3 — Description of Additional Supplementary Files [file 41467_2023_44654_MOESM3_ESM.pdf]

Description of Additional Supplementary Files

File Name: Supplementary Data 1
Description: Primers used in this study

File Name: Supplementary Data 2
Description: *S. cerevisiae* strains used in this study.

File Name: Supplementary Data 3
Description: Plasmids used in this study.

File Name: Supplementary Data 4
Description: Primers used in this study.

File Name: Supplementary Data 5
Description: Synthesized genes used in this study.
